# A toxicogenomic approach for the risk assessment of the food contaminant acetamide

**DOI:** 10.1016/j.taap.2019.114872

**Published:** 2020-02-01

**Authors:** Rance Nault, Bryan Bals, Farzaneh Teymouri, Michael B. Black, Melvin E. Andersen, Patrick D. McMullen, Seetha Krishnan, Nagesh Kuravadi, Neetha Paul, Santhosh Kumar, Kamala Kannan, K.C. Jayachandra, Lakshmanan Alagappan, Bhavesh Dhirajlal Patel, Kenneth T. Bogen, Bhaskar B. Gollapudi, James E. Klaunig, Tim R. Zacharewski, Venkataraman Bringi

**Affiliations:** a Institute for Integrative Toxicology, Biochemistry & Molecular Biology, Michigan State University, East Lansing, MI, United States of America; b Michigan Biotechnology Institute, Lansing, MI, USA; c ScitoVation, Durham, NC, USA; d Syngene International, Bengaluru, India; e Eurofins Advinus, Bengaluru, India; f ktbogen.com, Silver Spring, MD, USA; g Exponent, Alexandria, VA, USA; h Indiana University Bloomington, Bloomington, IN, USA; i Chemical Engineering & Materials Science, Michigan State University, East Lansing, MI, USA

**Keywords:** Toxicogenomics, Acetamide, Risk assessment, Cancer, Liver

## Abstract

Acetamide (CAS 60-35-5) is detected in common foods. Chronic rodent bioassays led to its classification as a group 2B possible human carcinogen due to the induction of liver tumors in rats. We used a toxicogenomics approach in Wistar rats gavaged daily for 7 or 28 days at doses of 300 to 1500 mg/kg/day (mkd) to determine a point of departure (POD) and investigate its mode of action (MoA). Ki67 labeling was increased at doses ≥750 mkd up to 3.3-fold representing the most sensitive apical endpoint. Differential gene expression analysis by RNA-Seq identified 1110 and 1814 differentially expressed genes in male and female rats, respectively, following 28 days of treatment. Down-regulated genes were associated with lipid metabolism while up-regulated genes included cell signaling, immune response, and cell cycle functions. Benchmark dose (BMD) modeling of the Ki67 labeling index determined the BMD_10_ lower confidence limit (BMDL_10_) as 190 mkd. Transcriptional BMD modeling revealed excellent concordance between transcriptional POD and apical endpoints. Collectively, these results indicate that acetamide is most likely acting through a mitogenic MoA, though specific key initiating molecular events could not be elucidated. A POD value of 190 mkd determined for cell proliferation is suggested for risk assessment purposes.

## Highlights

• Toxicogenomics can be used as weight-of-evidence to derive PODs for risk assessment.
• Acetamide's POD for cell proliferation was 190 mg/kg/day.
• Acetamide's rodent cancer induction is not consistent with other rodent carcinogens.
• There is strong concordance between transcriptional and apical PODs.

## 1 Introduction

Acetamide (CAS 60-35-5) is found in a wide range of human foods such as milk, eggs, beef, chicory roots, and roasted coffee beans (Vismeh et al., 2018). It is produced during food processing (Vismeh et al., 2018), as a metabolite of the pharmaceutical acetohydroxamic acid (AHA, sold as Lithostat®) prescribed to children with bladder infections (Bercu et al., 2018), and endogenously, likely through hepatic metabolism (Bogen et al., 2019). The International Agency for Research on Cancer (IARC) has classified acetamide as a Group 2B possible human carcinogen (IARC, 1999). This classification is largely based on the presence of liver tumors in chronically exposed rats as early as 1 year following initiation of exposure, and a modest increase in malignant lymphomas in male mice at high doses (≥1000 mg/kg/day (mkd) and ≥3000 mkd, respectively) (Dessau and Jackson, 1955; Weisburger et al., 1969; Fleischman et al., 1980; Flaks et al., 1983; Dybing et al., 1987). While the increased incidence of lymphomas is believed to be due to increased susceptibility to a mouse-specific virus (Bercu et al., 2010), little is known about the relevance of acetamide liver tumorigenesis to human health or its potential mode of action (MoA) in rats.

The genotoxicity of acetamide has been evaluated using numerous *in vivo* and *in vitro* assays, most of which were negative for genotoxicity. The Food and Agriculture Organization of the United Nations and World Health Organization (FAO/WHO) Joint Expert Committee on Food Additives (JECFA) declined to evaluate acetamide as a food flavoring agent in 2005 due to possible genotoxicity suggested by a positive mouse *in vivo* micronuclei (MN) assay reported by Chieli et al. (1987) (Abbott et al., 2006). However, those *in vivo* MN assay results could not be replicated, including when it was performed following Organization for Economic Co-operation and Development (OECD) guidelines (TG 471) and GLP standards in both mice and rats (Miura et al., 1994; Mirkova, 1996; Morita et al., 1997; De Boeck et al., 2005; Moore et al., 2019). Additionally, acetamide was not considered to be a mutagen based on the rat *in vivo* Pig-a gene mutation assay (Moore et al., 2019). Collectively, these studies indicate acetamide is non-genotoxic and the dose-response for tumors should be non-linear.

The non-genotoxic MoA for acetamide has been investigated with little success. Weisburger et al. (1969) hypothesized that ammonia produced by acetamide metabolism in chronically treated rats was responsible for carcinogenesis. While rats simultaneously fed acetamide and arginine glutamate, shown to prevent ammonia toxicities, did not develop liver tumors, equimolar administration of ammonium citrate also did not produce liver tumors suggesting that ammonia is not related to the MoA. Dybing et al. (1987) investigated the bioactivation of acetamide into AHA, a genotoxic metabolite, and incorporation into macromolecules. Using ^14^C-acetamide, <0.07% of acetamide was converted to AHA in either *in vitro* or *in vivo* models. Furthermore, acetamide did not covalently bind to proteins in primary hepatocytes, though treatment with cycloheximide reduced the level of ^14^C-acetamide and ^14^C-acetate incorporated into a non-extractable fraction suggesting incorporation into intermediary metabolism. Other studies have similarly reported negligible metabolism of acetamide to AHA (Putcha et al., 1984; Bogen et al., 2019). Consequently, the MoA of acetamide remains elusive.

We demonstrated that ammonia fiber expansion (AFEX), an emerging technology that increases the digestibility of crop residues and could improve ruminant livestock health and productivity, produces acetamide as a byproduct that is detectable in milk (Bals et al., 2010; Bals et al., 2019). This contamination of milk and other underappreciated levels in foods highlight the need to obtain further information regarding the relevance of rat carcinogenicity findings to human health (Vismeh et al., 2018). In the studies reported here, a toxicogenomics approach was used as an alternative strategy to chronic animal bioassays, to support assessments of the point of departure (POD) and attempt to uncover the MoA of acetamide (Thomas et al., 2012; Bhat et al., 2013; Farmahin et al., 2017; Farmahin et al., 2019; NTP, 2018). In toxicogenomics, differential gene expression is assumed to precede the emergence of apical effects and is considered a sensitive evaluation endpoint, and a powerful alternative for evaluating chemical safety. Proof-of-concept studies, examining ≥45 unique chemical compounds, demonstrate excellent concordance between PODs derived from chronic bioassays and acute/subchronic toxicogenomic evaluations (Thomas et al., 2007, 2011, 2012; 2013a,b; Bhat et al., 2013; Moffat et al., 2015; Webster et al., 2015; Labib et al., 2016; Farmahin et al., 2017; Farmahin et al., 2019; Kawamoto et al., 2017; Zhou et al., 2017; NTP, 2018). While most toxicogenomic studies have used a retrospective approach to examine the correlation between transcriptomic derived PODs with those derived from chronic animal studies, our study represents a case in which the hazard (tumorogenesis) is known, the MoA is poorly understood, and there is insufficient information to derive a POD. We show that toxicogenomic evaluation of acetamide can be used to identify a reference point for use in risk assessment that is also consistent with PODs for apical effects, *i.e.*, cell proliferation.

## 2 Materials and methods

### 2.1 Animals and treatment

The studies were conducted in an AAALAC accredited laboratory. For all experiments rats were acclimated for 5 days at 22 ± 3 °C with relative humidity of 30–70% and a 12 hours light:dark cycle at Eurofins Advinus Limited (Bengaluru, Karnataka, India). After acclimation, rats were randomly assigned to experimental groups using Provantis software (8.7.3, Instem LSS, Staffordshire ST15OSD, United Kingdom) for housing in sterilized polysulfone cages with stainless steel top grills with 2 rats per cage. Animals were fed Teklad Certified (2014C/2018C) Global 14%/18% Protein Rodent Maintenance Diet (Envigo, WI) and had access to water and food *ad libitum*. All procedures were performed in compliance with the guidelines provided by the Committee for Purpose of Control and Supervision of Experiments on Animals (CPCSEA) India, and were approved by the Institutional Ethics Committee (IAEC) of Eurofins Advinus Limited. Final reports are deposited in the open access Harvard Dataverse repository (dataverse.harvard.edu/dataverse/acetamide).

Experiment A was a dose range finding study in male Wistar Rats (Envigo, Israel; *N* = 6) aged 9–10 weeks orally gavaged daily with 30, 100, 300, or 1000 mg/kg/day (mkd) acetamide or water vehicle control (10 mL/kg) for 7 days. Male rats, the most sensitive sex for cancer (Fleischman et al., 1980) were used in this experiment. On day 8, following an overnight fast, animals were anesthetized under isoflurane and blood collected from the retro orbital plexus into tubes containing K_2_EDTA. Plasma was obtained by centrifugation at 5000 rpm at 2–8 °C for 5 min within 30 min of collection. Upon sacrifice, livers were weighed, and a sample (20–30 mg) of the left lobe was snap frozen in liquid nitrogen and stored at −70 °C for RNA-seq analysis. Other samples were fixed in 10% neutral buffered formalin (NBF) for histological evaluation. Supplementary Table S1, Supplementary Table S2 provide a complete list of tissues/endpoints weighed and/or collected. For urine collection, rats were individually housed in specially fabricated cages for 3–4 h with free access to water.

Experiment B was based on results from Experiment A and included male and female Wistar rats (Charles Rivers Laboratories, USA; N = 6) aged 8–10 weeks gavaged daily with 300, 500, 750, 1000 or 1500 mkd acetamide or water vehicle control (10 mL/kg) for 7 or 28 days. On days 8 and 29, 24 h following administration of the final dose, plasma, urine, and tissue samples were collected as described for experiment A. Animals were euthanized in a randomized sequence to minimize impact of circadian rhythmicity. Experiment B included a satellite treatment group consisting of 3 rats which were intraperitoneally injected with 5-bromo-2′-deoxyuridine (BrdU; 50 mg/kg) 5 consecutive days prior to sacrifice. Due to clear evidence that BrdU administration influenced cell proliferation endpoints (*e.g.*, increased Ki67 labeling ~3-fold in all groups including vehicle; Fig. S1), these are not discussed further but data is provided with the final study reports (dataverse.harvard.edu/dataverse/acetamide).

### 2.2 Hematology, clinical chemistry, & urinalysis

Hematological parameters were determined using an ADVIA 2120i hematology system (Siemens Healthcare Diagnostics Inc., NY, USA). For coagulation evaluation, samples were centrifuged at 2500 ×*g* for 10 min and analyzed using the Start Max/ Start-4 coagulation analyzer (Diagnostica stago, 92,600 Asnieres, France). Clinical chemistry parameters were measured using a Dimension RxL MaX Clinical Chemistry System (Dade Behring Inc. Newark, DE, USA) or Roche/Hitachi 902 (Hitachi High-Technologies Corporation, Tokyo, Japan). Urine volume was manually recorded, and microscopically evaluated for crystals, epithelial cells, erythrocytes, leukocytes, and casts. Urine parameters were measured by refractometry (Refractometer-PAL-10S, Atago Co., Ltd. Japan), Multistix 10 SG strips with Clinitek status analyzer (Bayer Healthcare LLC, UK), or Clinitek Advantus analyzer (Siemens Healthcare Diagnostics Inc., USA).

### 2.3 Immunohistochemistry

Formalin-fixed paraffin embedded liver sections (4–5 μm) were stained with Mayer's Hemotoxylin and Eosin. The mitotic index was determined by calculating the percent of hepatocytes in mitosis in 10 randomly selected 40× fields for a total of 3000 total hepatocytes. Liver sections were also stained for Ki67 using anti-Ki67 [SP6] antibody (Abcam; ab16667) at a 1:200 dilution and detected using biotinylated secondary goat anti-rabbit IgG (Vector Lab; PK-6101) at a 1:1000 dilution. An animal matched intestinal section was used as positive control. The Ki67 labeling index was determined by counting the number of positive and total hepatocytes in 10 randomly selected 40× fields similarly to the mitotic index for a total of 3000 hepatocytes.

### 2.4 Benchmark dose-response modeling of apical markers

PODs for apical markers (*e.g.*, cell proliferation and hepatocyte damage) were assessed by benchmark dose-response modeling in accordance with the European Food Safety Authority guidelines (Hardy et al., 2017) using the PROAST software (v66.16; https://www.rivm.nl/en/proast). We used the benchmark dose approach as it is now widely used by regulators to derive safe doses due to its advantages compared to other strategies such as the estimation of the no-observed-adverse-effect levels (Haber et al., 2018). Complete reports of BMD analyses are available in our Harvard Dataverse repository (dataverse.harvard.edu/dataverse/acetamide). Time-point and sex were used to generate a single covariate named “study” and a benchmark response (BMR) of 10% was selected considering the effect size and within-group variation (Slob, 2017). The BMDL_10_ and BMDU_a10_ for exponential and Hill family models were estimated as the lower and upper limits on the BMD 90% confidence interval, respectively, and the reported BMDL_10_ represents the lowest of the two model families. The PROAST software following EFSA guidance documents was used due to its capability of incorporating covariates.

### 2.5 Total RNA isolation and sequencing

Total RNA from the left liver lobe was isolated using the Maxwell RSC Simply RNA Tissue Kit (Promega, LOC). RNA was quantitated using the Qubit Broad Range Assay Kit (ThermoFisher, US), and quality was determined using the Agilent 4200 TapeStation. Libraries were prepared using the TruSeq Stranded mRNA Library Preparation Kit (Illumina, US) and were quantitated by Qubit (ThermoFisher) and assessed for quality by TapeStation (Agilent, US). Libraries were sequenced for 75 bp single-end reads at a depth of ~30 M/sample using the NextSeq 500 (Illumina).

RNA-seq reads were assessed for quality using FASTQC (www.bioinformatics.babraham.ac.uk/projects/fastqc/) and trimmed using Trimmomatic (Bolger et al., 2014) to remove the leading 7 bp and trailing 3 bp. Any reads <50 bp were rejected. Trimmed reads were aligned to the UCSC Rn6 indexed *Rattus norvegicus* reference genome using BowTie2 (Langmead and Salzberg, 2012). The number of reads mapping to Rn6 gene models was counted using HTSeq (Anders et al., 2015). Gene counts were analyzed for differential expression in R (v.3.4.3) using DESeq2 (v.1.20.0) (Love et al., 2014). A |fold change| ≥ 1.5 and FDR ≤ 0.05 threshold criteria was used to identify differentially expressed genes (DEGs). Raw reads and gene counts are deposited in the Gene Expression Omnibus (GEO; GSE132815).

### 2.6 Transcriptomics functional enrichment and dose-response modeling

MoAviz software looked at statistical over-representation analysis of DEGs among Biological Processes from the Gene Ontology consortium (www.geneontology.org) (McMullen et al., 2014, 2019; Pendse and McMullen, 2014). Enrichr and the ChEA database, derived from publicly available ChIP-seq, ChIP-chip, and gene expression profiling data (Lachmann et al., 2010), were used for transcription factor regulation predictions for the 500 genes with largest fold changes (Lachmann et al., 2010; Kuleshov et al., 2016).

Benchmark dose-response modeling of expression data was performed using BMDExpress2 (Phillips et al., 2019). Briefly, each gene was fit to Hill, power, linear, and polynomial (second- and third-degree) dose response models and the best fit model was identified using a hierarchical approach as outlined in the study report (dataverse.harvard.edu/dataverse/acetamide). Only genes with a raw sequence count >0 in all replicates and doses were considered to avoid bias in model fitting and linear models excluded. A BMR of 1.349 standard deviations, equivalent to a 10% change relative to control expression, and the 95% lower (BMDL_t_) and upper (BMDU_t_) confidence limits, were used for analysis of the transcriptomic data. Poor gene model fits were further filtered by excluding models with a goodness of fit *p*-value ≤ .1, a BMDU/BMDL ratio ≥ 40, a BMD value > than the highest tested dose, and a |fold change| < 1.5. Genes passing filtering criteria were subsequently matched to ontology elements and pathway/functional enrichment was determined when Fisher's exact test *p*-value ≤ .1 and membership ≥10 genes. 13 different pathway-agnostic and pathway-based approaches, outlined in Table S3, were used to aggregate genes for the estimation of POD to evaluate robustness of estimates as previously demonstrated (Farmahin et al., 2017).

Additionally, we used a novel approach of identifying the dose at which a single gene is expected to reach a specific effect size to provide weight of evidence to other POD estimates. Unlike benchmark dose-response modeling, this approach looks for the dose at which a single occurrence is observed rather that where it departs significantly from the control value. The number of DEGs with *K*-fold change were fit with a corresponding Hill dose-response model of the form Count = 1000/[1 + (*d*50/Dose)^*n*] estimating Hill exponent *n* and slope parameter *d*50, by iteratively re-weighted regression using inverse variance weights assuming Poisson-distributed count weights. The dose D1 at which less than a single gene is predicted to be differentially expressed was used as analogous to BMD. The BMDL analog is then the lower 1-tail 95% confidence bound (D1*) on D1. Each D1* value was calculated by simulating each *K*-specific data set assuming Poisson error about the corresponding fit obtained at each dose and *K*-value using the inverse of the original three fitted responses as corresponding sets of weights. The weighted fit to each set of simulated responses is then inverted to solve for dose D1 at which response is predicted to be 1, and a set of 10,000 such D1 estimates is then sorted and its 5th percentile value is taken to estimate D1*. This analysis was performed in *Mathematica*® 11.3 (Wolfram Research, Champaign, IL) as documented in supplemental materials.

### 2.7 Statistical evaluations

Data for animal studies was collected using Provantis (Instem, Staffordshire, UK) and statistically analyzed using built-in functions or the excel SYSTAT statistical package (v.12.0; Systat Software Inc., San Jose, CA, USA). Prior to running tests, data were evaluated for normality using the Shapiro-Wilk test and homogeneity of variances by Levene's method. Non-normally distributed or heteroschedastic data were appropriately transformed. All endpoints except gene expression were tested by one-way ANOVA followed by Dunnett's *post-hoc* test. Differences were considered significant when *p* ≤ .05. Fisher exact test for enrichment of individual gene lists was performed in R (3.4.1; www.r-project.org/). Multiple testing corrections were not used to assess significance – a practice consistent with standard rodent bioassay evaluations.

## 3 Results

### 3.1 Dose-range finding study

Previous acetamide bioassays have examined carcinogenesis at high doses (≥1000 mkd). Our dose range finding study (experiment A) was performed using 30, 100, 300 and 1000 mkd acetamide administered by daily gavage for 7 days. The study found no treatment-related changes in any of the measured endpoints at doses ≤300 mkd (Table S1). At 1000 mkd there was a decrease in absolute (25%) and relative liver weight (20%) (Table 1). Histology identified increased single-cell necrosis, minimal hepatocyte vacuolation, and a 4.2-fold increase in the Ki67 labeling index in the liver at 1000 mkd (Table 2). It is important to note that the term “single cell necrosis” has come under a re-examination in pathology evaluations since the pathology seen is more consistent with apoptosis (Elmore et al., 2016). The observed “single cell necrosis” in the current study showed hepatocytes with cytoplasmic and nuclear condensation and nuclear fragmentation. The plasma membrane was intact and there was no observed inflammation. This is consistent with an apoptotic *versus* a necrotic pathway. Treatment also increased plasma ALT (2.2-fold), AST (2.0-fold), ALP (1.6-fold), and total bilirubin (1.4-fold), and decreased plasma triglycerides 1.5-fold (Table 3 and Table S1). Absence of effects at low doses was consistent with negligible differential gene expression at doses ≤300 mkd, while 2685 genes were differentially expressed at 1000 mkd (Table 4). Consequently, 300 mkd was determined to be the no-observed-effect level (NOEL) and was therefore used as the lowest dose in the second experiment (experiment B). The remainder of the manuscript focuses on this second experiment.

### 3.2 Gross pathology & histopathology

Daily gavage of acetamide for 28 days did not affect terminal fasting body weight or body weight gain in either sexes or any dose group up to the highest tested dose of 1500 mkd (Table 1). No clinical signs, mortalities, or changes in food and water consumption were observed in any of the groups. An 11% decrease in relative liver weight was seen in male rats at 1500 mkd acetamide on day 8. There was also a 30% decrease in female adrenal gland weights at 1500 mkd on day 8 but not day 29, as well as a 50% decrease in male thymus weight at 1500 mkd on day 29 which was not significant when normalized to body weight (Table S2). These responses were inconsistent and do not appear to be related to treatment.

**Fig. 1:**
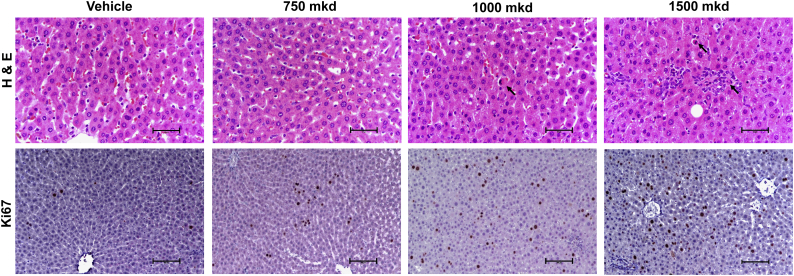
Representative photomicrographs of histological changes (H&E) and Ki67 labeling in livers of acetamide treated rats. Livers of female rats gavaged daily for 28 days are shown. Arrows indicate cells characterized as undergoing single-cell necrosis or apoptosis. Scale bar = 50 μm for H&E stained sections and 100 μm for Ki67 labeled sections.

H&E stained sections revealed single-cell necrosis categorized as “minimal”, and vacuolization in both male and female livers with neither feature exhibiting zonal distribution (Fig. 1). Again as noted above the histopathologic term “single cell necrosis” in the present study could be better classified as apoptosis. The Ki67 labeling index increased 2.1-fold in 1500 mkd treated males on day 8, and 3.3-fold at 1500 mkd for male rats on day 29 (Table 2). In one 1500 mkd treated male rat (Rv2655), Ki67 was induced ~5-fold higher than all other rats (37.5% *vs.* 7.5%). Grubb's test identified it as an outlier and there were no increases in other liver injury related endpoints (ALT, AST, Mitotic Index, liver weight) for this animal. In females, the Ki67 labeling index increased at doses ≥750 mkd up to 3.0-fold and 2.6-fold at days 8 and 29 days, respectively (Table 2). While a dose-dependent increase was observed at day 29, Ki67 labeling at day 8 was not dose-dependent with a 3.0-, 3.0-, and 2.3-fold increase at 750, 1000, and 1500 mkd, respectively.

**Table 1:** Changes in body and tissue weights in Wistar rats following daily oral gavage with acetamide.

Dose (mg/kg/day)	Terminal fasting body weight (g)	Liver weight (g)	Relative liver weight (% Body Weight)
Experiment A			
Males, day 8			
0	314.9 ± 8.2	9.77 ± 0.37	3.10 ± 0.09
30	316.3 ± 10.0	10.17 ± 0.26	3.22 ± 0.06
100	313.7 ± 9.5	9.48 ± 0.35	3.02 ± 0.07
300	313.0 ± 9.3	9.71 ± 0.49	3.10 ± 0.10
1000	293.7 ± 6.5	▼7.29 ± 0.24^a^	▼2.48 ± 0.08^a^

Experiment B			
Males, day 8			
0	294.3 ± 13.5	9.00 ± 0.50	3.06 ± 0.09
300	298.7 ± 8.1	9.23 ± 0.33	3.10 ± 0.12
500	301.9 ± 8.9	8.70 ± 0.41	2.88 ± 0.08
750	301.3 ± 6.7	9.00 ± 0.33	2.99 ± 0.07
1000	305.4 ± 10.1	8.98 ± 0.37	2.94 ± 0.07
1500	295.7 ± 6.3	8.04 ± 0.31	▼2.72 ± 0.06^a^
Males, day 29			
0	389.8 ± 9.1	11.23 ± 0.57	2.88 ± 0.10
300	384.7 ± 14.0	11.44 ± 0.45	2.98 ± 0.09
500	375.6 ± 12.4	10.76 ± 0.52	2.86 ± 0.09
750	379.2 ± 14.9	10.67 ± 0.51	2.81 ± 0.07
1000	383.8 ± 14.1	9.86 ± 0.43	2.57 ± 0.09
1500	362.3 ± 10.5	9.89 ± 0.34	2.73 ± 0.09
Females, day 8			
0	207.6 ± 4.0	6.05 ± 0.27	2.91 ± 0.11
300	210.5 ± 5.7	6.18 ± 0.28	2.93 ± 0.09
500	207.3 ± 4.3	5.69 ± 0.27	2.74 ± 0.09
750	204.3 ± 4.1	5.69 ± 0.18	2.78 ± 0.04
1000	201.0 ± 4.4	5.59 ± 0.30	2.78 ± 0.12
1500	202.5 ± 5.1	5.92 ± 0.28	2.92 ± 0.08
Females, day 29			
0	245.2 ± 9.7	6.46 ± 0.34	2.63 ± 0.07
300	236.2 ± 5.4	6.35 ± 0.14	2.69 ± 0.07
500	235.8 ± 9.7	6.27 ± 0.26	2.66 ± 0.05
750	234.5 ± 6.4	5.90 ± 0.20	2.52 ± 0.07
1000	231.8 ± 7.1	5.67 ± 0.17	2.45 ± 0.06
1500	224.8 ± 5.1	5.91 ± 0.10	2.63 ± 0.05

a Significant difference (*P* ≤ .05) compared to vehicle control animals determined by one-way ANOVA and Dunnett's *post-hoc* test using Provantis.

**Table 2:** Histological evaluation of livers from Wistar rats following daily oral gavage with acetamide.

	Histopathology (incidence in 6 rats)					Cell proliferation	
Dose (mg/kg/day)	Vacuolation			Mononuclear cell infiltration	Single-cell necrosis		Ki67 labeling index (%)^a^
	Minimal	Mild	Moderate	Minimal	Minimal	Mild	
Experiment A							
Males. Day 8							
0	0	0	0	0	0	0	5.89 ± 1.01
30	0	0	0	0	0	0	▼2.93 ± 0.65^b^
100	0	0	0	0	0	0	▼2.56 ± 0.46^b^
300	0	0	0	0	0	0	4.31 ± 0.70
1000	5	0	0	0	3	2	▲24.62 ± 3.58^b^

Experiment B							
Males, day 8							
0	0	0	0	0	0	0	2.81 ± 1.75
300	1	2	1	0	0	0	2.42 ± 1.42
500	2	1	0	1	0	0	3.16 ± 1.42
750	1	0	0	0	0	0	4.15 ± 1.84
1000	1	0	0	2	1	0	4.26 ± 1.69
1500	1	1	0	0	4	0	▲5.93 ± 1.45^b^
Males, day 29							
0	0	0	1	0	0	0	3.14 ± 0.76
300	0	0	1	0	0	0	2.38 ± 0.75
500	3	2	0	1	0	0	3.72 ± 0.87
750	1	2	0	0	0	0	3.87 ± 0.86
1000	1	0	0	0	2	0	4.19 ± 0.71
1500	2	0	2	1	2	0	▲10.31 ± 13.35^b^
Females, day 8							
0	0	0	0	1	0	0	1.18 ± 0.44
300	0	0	0	0	0	0	1.58 ± 1.19
500	0	0	0	0	0	0	1.87 ± 0.91
750	0	0	0	0	0	0	▲3.53 ± 1.74^b^
1000	2	0	0	0	2	0	▲3.51 ± 1.75^b^
1500	3	0	0	0	4	0	▲2.73 ± 0.64^b^
Females, day 29							
0	0	0	0	1	0	0	2.23 ± 0.81
300	0	0	0	0	0	0	2.66 ± 1.11
500	0	0	0	0	0	0	2.75 ± 0.64
750	0	0	0	0	0	0	▲4.09 ± 0.28^b^
1000	1	0	0	0	1	0	▲4.58 ± 1.14^b^
1500	4	0	0	0	4	0	▲5.69 ± 0.95^b^

a A total of 3000 hepatocytes were counted.

b Significant difference (*P* ≤ .05) compared to wild-type control determined by one-way ANOVA and Dunnett's *post-hoc* test.

**Table 3:** Changes in plasma clinical chemistry in Wistar rats following daily oral gavage with acetamide.

Dose (mg/kg/day)	AST (U/L)	ALT (U/L)	Total bilirubin (μM)	Total cholesterol (mM)	Triglycerides (mM)
Experiment A					
Males, day 8					
0	76.67 ± 3.88	57.00 ± 4.46	3.22 ± 0.22	2.15 ± 0.11	1.29 ± 0.10
30	66.17 ± 3.92	56.67 ± 3.84	2.89 ± 0.19	2.28 ± 0.15	1.51 ± 0.16
100	69.67 ± 1.84	52.83 ± 4.19	3.25 ± 0.18	1.93 ± 0.11	1.13 ± 0.19
300	▲78.67 ± 7.02^a^	▲58.83 ± 4.45^a^	▼2.17 ± 0.35^a^	2.25 ± 0.13	0.81 ± 0.14
1000	▲167.83 ± 25.70^a^	▲114.67 ± 12.94^a^	4.61 ± 0.82	2.44 ± 0.09	▼0.68 ± 0.12^a^

Experiment B					
Males, day 8					
0	79.17 ± 2.30	42.83 ± 2.23	2.94 ± 0.18	2.22 ± 0.11	0.68 ± 0.10
300	73.83 ± 4.81	43.00 ± 5.47	2.92 ± 0.24	2.11 ± 0.25	0.79 ± 0.18
500	77.00 ± 1.44	47.50 ± 2.7	3.18 ± 0.14	2.68 ± 0.14	0.79 ± 0.10
750	78.00 ± 2.21	52.67 ± 3.3	2.92 ± 0.25	2.48 ± 0.09	0.65 ± 0.09
1000	80.67 ± 4.45	55.00 ± 6.77	2.73 ± 0.39	2.75 ± 0.12	0.82 ± 0.12
1500	▲99.67 ± 4.70^a^	55.67 ± 5.71	2.99 ± 0.23	2.72 ± 0.15	0.97 ± 0.13
Males, day 29					
0	74.67 ± 4.36	44.00 ± 2.41	2.24 ± 0.27	1.95 ± 0.12	0.45 ± 0.07
300	74.50 ± 5.25	43.33 ± 1.58	2.25 ± 0.28	2.24 ± 0.17	0.41 ± 0.03
500	77.33 ± 5.45	47.50 ± 2.26	2.88 ± 0.38	2.10 ± 0.21	0.43 ± 0.06
750	63.33 ± 3.78	41.33 ± 2.29	2.89 ± 0.30	2.31 ± 0.13	0.55 ± 0.10
1000	93.00 ± 8.72	55.17 ± 6.33	3.03 ± 0.34	2.27 ± 0.08	0.51 ± 0.12
1500	96.67 ± 14.79	▲63.33 ± 8.77^a^	3.62 ± 0.85	2.70 ± 0.29	0.46 ± 0.07
Females, day 8					
0	83.17 ± 3.50	40.67 ± 1.38	2.80 ± 0.14	2.79 ± 0.13	0.98 ± 0.27
300	90.83 ± 1.83	40.67 ± 2.63	3.25 ± 0.49	2.66 ± 0.08	1.44 ± 0.24
500	94.33 ± 9.05	41.67 ± 2.56	3.49 ± 0.29	2.58 ± 0.11	0.94 ± 0.13
750	88.00 ± 3.70	43.83 ± 2.33	3.16 ± 0.31	2.95 ± 0.14	1.13 ± 0.17
1000	101.00 ± 6.85	▲51.17 ± 2.56^a^	2.90 ± 0.37	2.67 ± 0.06	0.67 ± 0.14
1500	95.67 ± 2.82	▲55.33 ± 4.32^a^	2.91 ± 0.22	2.99 ± 0.17	0.79 ± 0.16
Females, day 29					
0	80.50 ± 5.18	41.67 ± 3.68	2.55 ± 0.18	2.50 ± 0.13	0.41 ± 0.04
300	79.17 ± 4.71	36.83 ± 2.02	3.04 ± 0.33	2.33 ± 0.07	0.56 ± 0.06
500	87.00 ± 9.37	38.00 ± 2.52	3.27 ± 0.47	2.54 ± 0.09	0.50 ± 0.04
750	101.17 ± 16.68	44.00 ± 2.46	3.59 ± 0.48	2.52 ± 0.11	0.62 ± 0.08
1000	93.67 ± 6.23	47.83 ± 3.77	▲3.61 ± 0.22^a^	2.40 ± 0.11	0.59 ± 0.10
1500	136.83 ± 30.32	86.67 ± 31.79	▲4.88 ± 1.00^a^	2.98 ± 0.34	0.53 ± 0.09

a Significant difference (*P* ≤ .05) compared to wild-type control determined by one-way ANOVA and Dunnett's *post-hoc* test using Provantis.

**Table 4:** Differential gene expression (|fold change ≥1.5 & FDR ≤ 0.05) in Wistar rats following daily oral gavage with acetamide.

Regulation direction	Dose (mkd)							
	30	100	300	500	750	1000	1500	All doses
Experiment A								
Males, day 8								
Up	0	0	1	–	–	1717	–	2685
Down	0	0	0	–	–	968	–	

Experiment B								
Males, day 8								
Up	–	–	0	0	0	0	129	176
Down	–	–	0	1	0	0	47	
Males, day 29								
Up	–	–	1	1	62	222	394	1110
Down	–	–	1	21	237	418	341	
Females, day 8								
Up	–	–	0	6	146	1679	261	485
Down	–	–	1	1	79	80	80	
Females, day 29								
Up	–	–	0	52	109	352	1202	1814
Down	–	–	0	6	48	109	478

### 3.3 Hematology, urinalysis, & clinical chemistry

Supplementary Table S1, Supplementary Table S2 summarize the hematology, clinical chemistry, and urinalysis endpoints. Only plasma aspartate aminotransferase (AST), alanine aminotransferase (ALT), and total bilirubin (T.Bil) showed treatment related effects in experiment B (Table 3). ALT was increased up to 1.4-fold at the highest dose in females at day 8 and males at day 29. The highest dose in other treatment groups similarly caused a non-significant 1.3- and 2.1-fold increase in plasma ALT in males at day 8 and females at day 29, respectively. It should be noted that these fold-changes in serum ALT are small compared to many hepatotoxicants (Slob, 2017). Conversely, AST only increased 1.3-fold in males at 8 days at 1500 mkd, though 1.2- to 1.7-fold increases were observed in the other treatment groups. Increased total bilirubin was only observed at ≥1000 mkd in females at 29 days. Small changes in red cell distribution width (RDW), hypochromic cells (Hypo), red blood cell ghosts (RBC Ghosts), eosinophils, activated partial thromboplastin time (APTT), glucose, alkaline phosphatase (ALP), Albumin (Alb), hemoglobin (HBG), and hematocrit (HCT) were also observed, but not considered to be treatment related as they were either not dose-dependent, within historical control levels, or inconsistent between experimental groups.

### 3.4 Apical BMD (BMD_10_) modeling

**Fig. 2:**
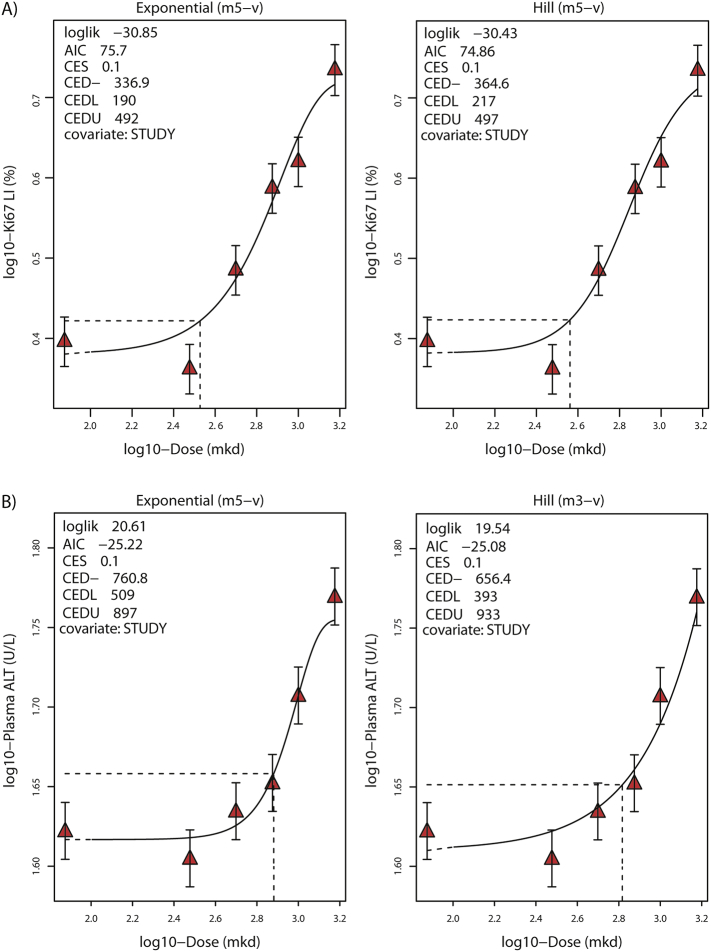
Benchmark dose-response modeling of apical endpoints (A) Ki67 labeling index (Ki67-LI) and (B) plasma ALT. Best fitted model for exponential (left panel) and Hill (right panel) model families were determined based on the lowest Akaike Information Criterion (AIC) using PROAST v66.16 in R. Red triangles represent mean values and whiskers show confidence interval. Dose is plotted on a log10-scale while the level of the control sample is placed at an arbitrary level lower than the lowest dose on the log-transformed x-axis, denoted by blue circles. The critical effect dose (CED, equivalent to BMD) and its lower and upper confidence limits (CEDL and CEDU, respectively, equivalent to BMDL and BMDU) were determined at a critical effect size (equivalent to BMR) or 10%.

Ki67 labeling in female rats, the most sensitive apical marker with a lowest-observed-effect level (LOEL) of 750 mkd (Table 2), was used for benchmark dose-response modeling in accordance with EFSA guidelines (Hardy et al., 2017). The small increases in ALT were also modeled to support the Ki67 BMD estimates. While large increases in ALT is a marker of hepatocyte damage, small increases are often observed with compounds which promote cell proliferation (Hall et al., 2012). Time and sex were combined as a variable termed “study” and used as covariates. Due to low Ki67 labeling in females on day 8, lack of dose response at doses ≥750 mkd, and unequal variance (Bartlett's test *p* = .04), this dataset was excluded from BMD modeling. The outlier rat (Rv2655) was also excluded. Dose-response modeling of the Ki67 labeling index calculated a BMDL_10_ of 190 mkd and BMDU_10_ of 497 mkd with a confidence interval (BMDU/BMDL) of 2.6 using the minimum and maximum values for both exponential and Hill family models (Fig. 2A). The ALT BMDL_10_ and BMDU_a_ were 393 and 933 mkd, respectively, with a confidence interval of 2.4 (Fig. 2B).

### 3.5 Gene expression changes

RNA-Seq analysis identified 2685 differentially expressed genes in the male rat dose range finding study (*experiment A*), all of which are differentially expressed at the highest dose (1000 mkd). Only *Hebp2* was also differentially expressed at a lower dose, showing up-regulation at 300 and 1000 mkd (Table S4). In *experiment B,* 176 and 485 differentially expressed genes (DEGs; |fold change| ≥ 1.5 & FDR ≤ 0.05) were identified in acetamide-treated male and female rats on day 8, respectively, while 1110 and 1814 DEGs were identified in male and female rats on day 29, respectively (Table 4; Supplementary Table S5, Supplementary Table S6, Supplementary Table S7, Supplementary Table S8). *Acot1* was among the most upregulated genes in both sexes and time-points (ranked 1 (4.94-fold), 15 (3.40-fold), 2 (5.06-fold), and 6 (4.55-fold) in males day 8, females day 8, males day 29, and females day 29, respectively) while *Ifit1* was the most induced in both sexes at 28 days (7.0- and 7.6-fold in males and females, respectively). Collectively, the magnitude of fold changes elicited by acetamide exposure was modest, where only 20% of differential expression at the highest dose of acetamide (1500 mkd) at day 29 was above 3-fold for both sexes and 50% between 1.5- and 2-fold (Fig. S1).

**Fig. 3:**
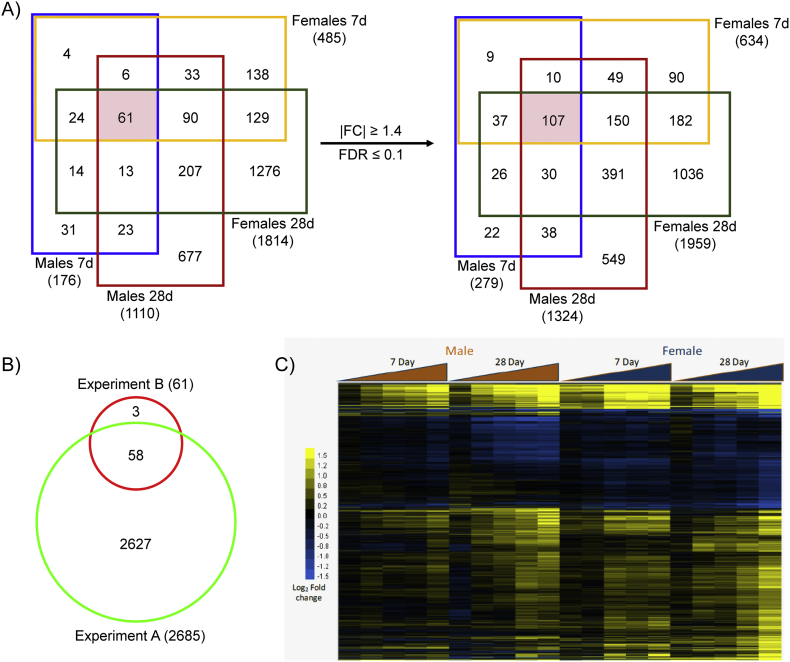
Comparison of acetamide elicited gene expression changes across treatment durations and sexes in experiment B. (A) DEGs identified by normal criteria (|fold change| ≥ 1.5 & FDR ≤ 0.05) were compared across datasets, as well as the union of these 2726 genes using relaxed criteria (|fold change| ≥ 1.4 & FDR ≤ 0.1). (B) DEGs common to all time-points and sexes in experiment B were compared to the 2685 DEGs from experiment A. (C) Cluster analysis of the union of DEGs at 1500 mkd. Genes are shown along the y-axis with increasing acetamide doses along the x-axis. Log2 fold change values were centered by gene to highlight dose-, time-, and sex-related differences.

Comparative assessment of DEGs from *experiment B* found a total of 61 DEGs common to all time-points and sexes. However, reducing the |fold change| and FDR criteria for the union of these 2726 genes (1.4 and 0.1, respectively) increased the overlap to 107, suggesting many genes approach the threshold criteria (Fig. 3A). Of the 61 genes common to all sexes and time-points, 58 were also differentially expressed in *experiment A* (Fig. 3B). The three genes (*Cenpw*, *Chek2*, and *Parpbp*) that did not overlap narrowly missed threshold criteria (Supplementary Table S4, Supplementary Table S5, Supplementary Table S6, Supplementary Table S7, Supplementary Table S8). Cluster analysis of the union of all DEGs at the highest dose in any treatment group (2152 genes) from *experiment B* exhibited similar expression profiles across treatment groups (Fig. 3C), further suggesting that differences are due to the modest changes elicited by acetamide. Only three genes, *Cyp17a1* (induced in females, repressed in males), *Slc7a1* (induced in females, repressed in males), and *Prom1* (induced in males, repressed in females), exhibited discordant expression.

**Fig. 4:**
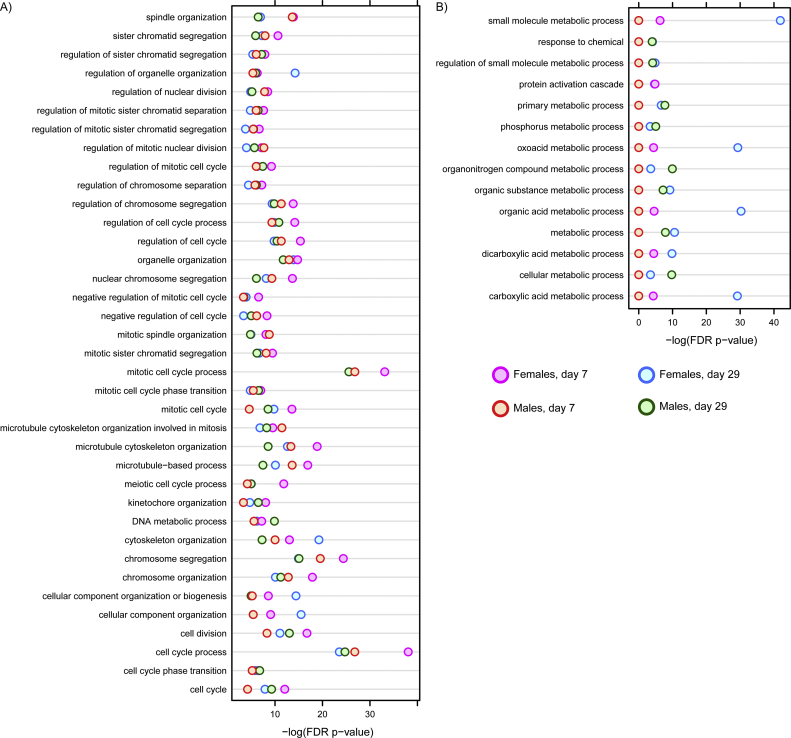
Functional enrichment analysis of differentially expressed genes (|fold change| ≥ 1.5 & FDR ≤ 0.05) in Wistar rats gavaged daily with acetamide. MoAviz was used to evaluate over-representation in GO ontologies for (A) up-regulated and (B) down-regulated genes. Enriched GO ontologies conserved in all treatment groups (experiment B) is shown for up-regulated genes while those enriched in at least 2 treatment groups are shown for down-regulated genes as no GO ontologies were conserved in all treatment groups.

**Fig. 5:**
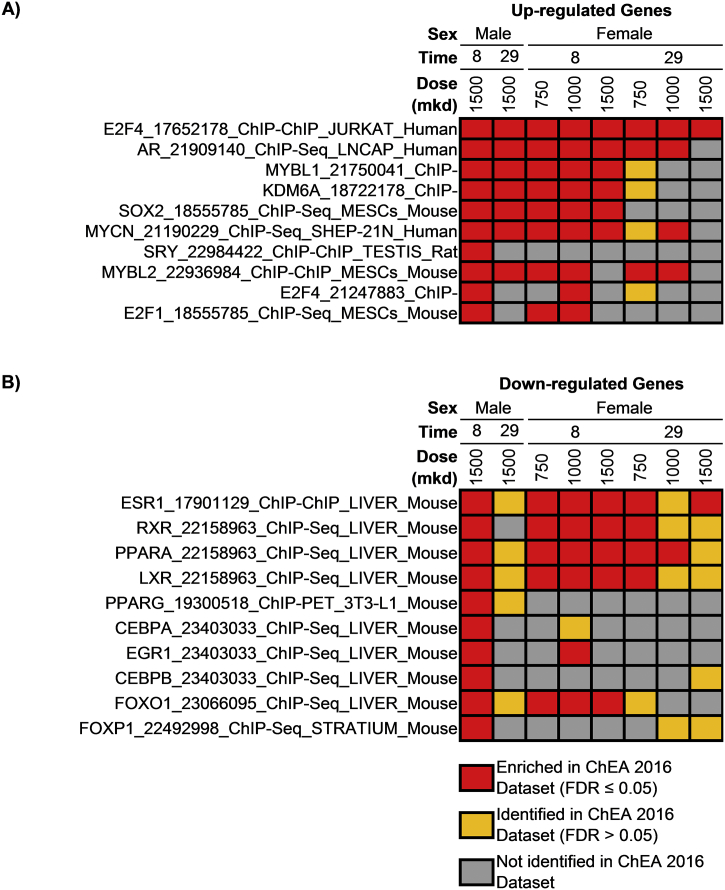
Enrichr analysis using the ChEA 2016 datasets for the 500 most (A) up-regulated and (B) down-regulated DEGs (|fold change| ≥ 1.5 & FDR ≤ 0.05) following acetamide exposure. Only doses at which increased cell proliferation was observed are included, and datasets identified in multiple doses and/or sexes are shown.

Functional enrichment analyses associated differential gene expression with cell cycle regulation and mitotic processes for induced genes which was consistent across all times and sexes (Fig. 4). Conversely, repressed genes were associated with metabolism of small molecules and amino acids. Enrichr (Lachmann et al., 2010) enrichment analysis associated transcriptional regulators E2F4, AR, MYBL1, KDM6A, and SOX2 with up-regulated genes while ESR1, RXR, PPARA, and LXR were associated with down-regulated genes (Fig. 5). These data suggest that acetamide exposure promotes cell cycle regulation and signaling, and represses various metabolic processes.

### 3.6 Transcriptional BMD (BMD_t_) modeling

BMD modeling of transcriptomic data was performed using BMDExpress2. In *experiment A*, the best fit to 37.4 and 34.6% of the genes were with the exponential 2 and power models, respectively, consistent with differential expression only at the highest dose. In *experiment B,* the best fits to 23%–39% and 13%–21% of the DEGs were with the exponential 2 and Hill models, respectively. Transcriptional POD using median BMDL_t_ values were estimated using ontology agnostic and ontology-based approaches that produce concordant estimates with apical endpoints assessed in chronic animal bioassays (Thomas et al., 2007, 2011, 2012; 2013a,b; Bhat et al., 2013; Moffat et al., 2015; Webster et al., 2015; Labib et al., 2016; Farmahin et al., 2017, Farmahin et al., 2019; Kawamoto et al., 2017; Zhou et al., 2017; NTP, 2018). Additionally, transcriptional PODs were derived separately for induced and repressed genes – the two groups that were clearly associated with distinct functions (cell cycle and metabolism, respectively).

**Fig. 6:**
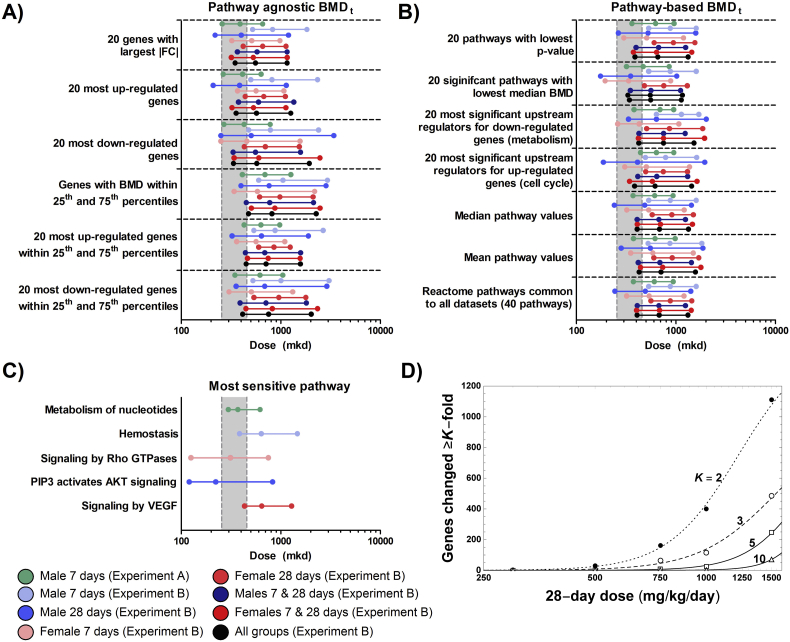
Estimation of acetamide’s BMD confidence interval using dose-response modeling of differential gene expression. The median BMDL_t_ – BMD_t_ – BMDU_t_, shown in order as three points on a horizontal line, were derived using several (A) pathway agnostic and (B) pathway-based approaches. (C) The BMDL_t_ – BMD_t_ – BMDU_t_ range is also shown for the most sensitive pathway in each study, time-point, and sex. BMDt values were determined using BMDExpress2 as described in the materials and methods. The gray boxes represent the BMDLa to BMDUa range for the Ki67 labeling index. (D) The dose-dependent number of DEGs (|fold change| ≥ K & FDR ≤ 0.05) was modeled, and its lower 1-tail 95% confidence bound (D1*, equivalent to BMDL) determined revealing D1* values of 225, 270, 478, and 843 mkd for a K-fold change of 2, 3, 5, and 10, respectively.

Ontology agnostic PODs ranged from 210 mkd to 615 mkd across all experiments, treatment durations, and sexes (Fig. 6A). Though functionally distinct, there was no difference on the PODs for the 20 most induced (210–501 mkd) or repressed (250–477 mkd) genes. Similarly, ontology-based approaches demonstrated PODs ranging from 175 mkd to 637 mkd (Fig. 6B). Consistent with neither up- or down-regulated genes demonstrating different PODs, no clear difference was observed for the 20 most significant up or down-regulated regulators. When examining enriched pathways common to all datasets, the average median BMDL_t_ was 407 mkd. The 8 pathways common to all datasets included signal transduction, metabolism, metabolism of protein, immune system, post-translational protein modification, hemostasis, cell cycle, and mitotic cell cycle. Among these common enriched pathways, the most sensitive (post-translational protein modification, females day 8) had a POD of 167 mkd. Conversely, the pathways with the lowest median BMDL_t_ values in each dataset individually were metabolism of nucleotides (297 mkd, experiment A), hemostasis (385 mkd, males day 8), signaling by Rho GTPases (125 mkd, females day 8), PIP3/AKT signaling (120 mkd, males day 29), and signaling by VEGF (431 mkd, females day 29) (Fig. 6C). Most importantly, the median BMDL_t_ – BMD_t_ – BMDU_t_ ranges using any of these approaches either spanned the BMD_10_ confidence interval for Ki67 labeling index (190–497 mkd) or were above the BMDU_a_ demonstrating concordance between PODs. Furthermore, modeling of the dose at which a single gene was altered by 2-, 3-, 5-, or 10-fold found that the BMDL equivalent (D1*) was 225, 270, 478, and 843 mkd, respectively (Fig. 6D).

## 4 Discussion

In the risk assessment of chemicals, regulators often rely on PODs derived from chronic animal bioassays as a reference point to make an informed decision regarding acceptable levels of exposure. However, chronic assays are resource-intensive and expensive, and often fail to provide information regarding the MoA (NTP, 2004; Thomas et al., 2012; EFSA, 2014; Chepelev et al., 2015). Furthermore, while regulatory agencies have committed to evaluating priority chemicals (Barton-Maclaren et al., 2017; Farmahin et al., 2017), it is clear that the current risk assessment paradigm cannot keep pace with the numbers of chemicals that need testing. In response, alternative approaches have been explored including the application of toxicogenomics (NTP, 2018; Yauk et al., 2019). In this study, we present a case scenario for the application of toxicogenomics in the risk assessment of acetamide.

### 4.1 Using toxicogenomics to derive a POD for use in risk assessment

Deriving a POD from acute and subchronic toxicogenomic studies is an ongoing debate between sponsors, stakeholders, and regulatory agencies. To date, toxicogenomic efforts have largely used a retrospective approach where chemicals which have been well characterized in chronic studies are evaluated by toxicogenomic and these PODs are contrasted to those from traditional apical endpoints. These studies have demonstrated that transcriptomic PODs derived using various approaches are consistent with PODs derived from traditional end points such as cell proliferation and tumor formation (Thomas et al., 2007, 2011, 2012; 2013a,b; Bhat et al., 2013; Moffat et al., 2015; Webster et al., 2015; Labib et al., 2016; Farmahin et al., 2017, Farmahin et al., 2019; Kawamoto et al., 2017; Zhou et al., 2017; NTP, 2018). In this study, 18 different approaches to estimate acetamide's POD from transcriptomic data were used. We found that the transcriptomic PODs were consistent (±3.5-fold) with PODs for increased cell proliferation (Ki67 labeling index), an endpoint considered biologically relevant for the development of tumors. Consistent with an evaluation of 11 different approaches to derive transcriptional PODs for 6 different known rodent carcinogens (Farmahin et al., 2017), acetamide median BMDL_t_ estimates for the 20 pathways with the lowest BMD_t_ (175 mkd) and for 20 genes with the largest fold-changes (219 mkd) were very similar to the apical POD for cell proliferation of 190 mkd.

Most notable in the transcriptomic evaluation was up-regulation of genes associated with cell cycle and cell proliferation. Many non-genotoxic carcinogens promote tumorigenesis by increasing the number of cells through increased cell proliferation and/or a decrease in cell death (apoptosis). The upregulation of genes associated with cell cycle is consistent with the most sensitive apical marker, Ki67 labeling index, with a POD of 190 mkd. In a recent report from the U.S. National Toxicology Program (NTP) on the use of transcriptomic data for chemical safety evaluations, it was noted that transcriptional PODs will need to be linked to more standard measurements for use in risk assessment (NTP, 2018). In our study, two cell cycle related pathways were identified among the most sensitive conserved pathways, “cell cycle” and “mitotic cell cycle”, which both had median BMDL_t_s of 185 mkd. The similarity of the POD estimates for both pathways is likely due to the redundancy in gene membership for these two ontologies. The similarity of the POD between both the apical marker and most sensitive conserved pathways suggests that a POD of 185–190 mkd may reflect the best estimate of the POD for acetamide. Further supporting the reliability of deriving a POD using this approach is the strong concordance of all other approaches where the BMDL_t_ – BMDU_t_ range either fell within or above the Ki67 labeling index BMDL_t_ – BMDU_t_ range. Collectively, the weight of evidence supports using the acute/subchronic apical marker POD of 190 mkd since it (i) can be anchored to the apical endpoint of cancer, (ii) is consistent with gene expression changes and their estimated PODs, and (iii) is supported by several other approaches for deriving a gene expression POD previously shown to be consistent with traditional toxicology assays.

### 4.2 Elucidation of acetamide's MoA

In the current risk assessment paradigm, toxicogenomics data have largely been used to provide insights and weight of evidence on the chemical MoA (Yauk et al., 2019). Our results demonstrate that acetamide increases cell proliferation which is consistent with the MoA for other non-genotoxic carcinogens (Cohen and Ellwein, 1990). However, the molecular initiating and key events through which acetamide promotes mitogenesis remain unclear (Weisburger et al., 1969; Flaks et al., 1983; Dybing et al., 1987). To further examine acetamide's MoA, we explored whether gene expression changes were consistent with known rodent liver carcinogen MoAs (Cohen, 2010) summarized in Table 5.

**Table 5:** Evidence for acetamide's activity through known rodent liver carcinogen modes of action.

Category	MOA/KIE	Evidence
Genotoxic/DNA reactive		No evidence of genotoxicity based on literature and absence of gene expression changes related to p53 signaling.
Non-genotoxic/non-DNA reactive		Yes. By default since not genotoxic

Receptor mediated		
	CAR (phenobarbital-like)	Not enriched among DEGs. No induction of known targets (*e.g. Cyp2b, Cyp3a11*).
	AhR (dioxin-like)	Not enriched among DEGs. No induction of known targets (*e.g. Cyp1a1, Cyp1a2*).
	PPAR alpha	Enriched among down-regulated genes, contrary to carcinogens acting through this MoA.
	Steroid hormone receptors	No evidence in DEGs. No evidence of endocrine disruption in the ToxCast screening database.

Non receptor mediated		
	Cytotoxicity	Apoptosis beginning at higher doses could promote regenerative proliferation.
		Cytotoxic *in vitro* at extremely high concentrations.
	Oxidative stress (OS)	Modest modulation (up and down) of glutathione related genes, repression of *Nqo1*. Unlikely to promote oxidative stress.
	Immune /inflammation response	Some evidence of immune cell signaling (GCPR, Rho GTPase, FGCR, and PD-1). No inflammation in histopathological evaluation here or in chronic bioassays (ref).
	Fatty liver/Steatosis	Minor vacuolation at high doses. No progression (steatohepatitis and fibrosis) in chronic bioassays (ref)

In agreement with *in vitro* and *in vivo* assays genotoxic (Miura et al., 1994; Mirkova, 1996; Morita et al., 1997; De Boeck et al., 2005; Moore et al., 2019), differential expression did not indicate genotoxicity when compared to results with known genotoxicants such as formaldehyde, naphthalene, chloroprene, and benzo[*a*]pyrene (Andersen et al., 2008, 2010; Thomas et al., 2013a; Clewell et al., 2014; Moffat et al., 2015). Although cell cycle related genes were induced, there was no evidence of p53 mediated DNA damage or oxidative stress pathways as found with genotoxic compounds (Watanabe et al., 2012). Consequently, mitogenic responses with acetamide may be mediated by receptor-mediated or by processes unrelated to these nuclear receptors (Holsapple et al., 2006; Cohen, 2010; Corton et al., 2014; Klaunig et al., 2015; Wang et al., 2015; Wang et al., 2017a,b). Receptor-mediated carcinogenesis typically involves the activation of nuclear transcription factors – such as CAR (constitutive androstane receptor), PPARα (peroxisome proliferator-activated receptor), AHR (aryl hydrocarbon receptor), ER (estrogen) or AR (androgen) receptors. However, functional enrichment analyses did not identify CAR-, AHR-, and AR-mediated activity. Curiously, the downregulated genes were highly enriched for binding by PPARα, ESR1 (ER), and LXR. Marker genes for CAR (*Cyp2b*, *Cyp3a11*) and AHR (*Cyp1a1*, *Cyp1a2*) were also unchanged by acetamide.

Possible non receptor-mediated MoAs for carcinogens include cytotoxicity, oxidative stress, immunotoxicity, and steatosis (Hernandez et al., 2009; Cohen, 2010). In a toxicogenomics evaluation of hexabromocyclododecane in rats, 18 genes were proposed to identify cytotoxic carcinogens (Farmahin et al., 2019). Only 5 were differentially expressed by acetamide including *Akr1b8* (Females day 8 & 29; up to 7.8-fold), *Rnd1* (Females day 8 & 29; up to 2.8-fold), *Tes* (Females day 29, Males day 29; up to 2.0-fold), *Car3* (Females day 8; repressed up to 5.9-fold), and *S100a10* (Males day 29; up to 2.0-fold). A Fisher's exact test did not indicate significant enrichment (*p* = .19). However, apoptosis which is observed at higher doses, could be associated with regenerative proliferation in the liver as suggested for fuminosin B_1_ which may lead to tumorogenesis (Dragan et al., 2001). Pathways related to oxidative stress were also not significantly enriched and known responsive genes were either repressed, such as *Nqo1* which was repressed 2.6-fold in females on day 29, or unchanged such as *Sod1*, *Sod2*, and *Gpx1*. *Gstp1* showed a 1.7-fold increase in males at 29 days, though this was only at the 1000 mkd dose. Despite enrichment of immune related pathways including G protein coupled receptors, Fc receptors, and PD-1 signaling, there was no evidence of immunotoxicity or inflammation (Dessau and Jackson, 1955; Weisburger et al., 1969; Flaks et al., 1983). Although minimal lipid vacuolation was observed, lipid metabolism related genes were repressed. The absence of inflammation and minimal fat accumulation does not support the progression of steatohepatitis to fibrosis/cirrhosis, and eventually hepatocellular carcinoma as a possible MoA for acetamide (Dessau and Jackson, 1955; Weisburger et al., 1969; Flaks et al., 1983).

While this study indicated that the MoA for acetamide is related to persistent mitogenic stimulation which precedes the occurrence of necrosis/apoptosis, it also highlighted differences between acetamide and other mitogenic compounds. For example, unlike other rodent carcinogens such as toxaphene (Wang et al., 2015) and 1,4-dichlorobenzene (Eldridge et al., 1992) that cause transient increases in cell proliferation and increased liver weight, acetamide-induced proliferation persisted throughout the 28-day study without any increase in absolute or relative liver weight. Absence of liver weight increase may be due to increased necrosis/apoptosis at higher doses. MAPK signaling was also enriched in BMD analyses which would be consistent with apoptosis.

Acetamide is an antidote to fluoroacetamide poisoning through competition for acetyl-CoA synthetase (Gitter, 1956; Goncharov et al., 2006; Proudfoot et al., 2006), and high-throughput screening detected inhibition of Histone Lysine Methyltransferase G9a activity (PubChem AID: 504332). Such interference with acetate/acetyl-CoA dependent enzymes at very high intracellular levels could potentially result in altered acetyl-CoA levels which can disrupt important cellular processes (Pietrocola et al., 2015). High levels of acetate-like metabolites, for example, could lead to the suppression of metabolic processes controlled by PPAR, LXR, and RXR. The ability of arginine-glutamate to prevent cancer development in acetamide-treated rats also suggests that the urea, glutathione, and TCA cycle metabolic pathways may be affected by acetamide exposures, though studies have shown that arginine glutamate may simply act as an inhibitor of cell proliferation through induction of polyamine synthesis (Takeda et al., 1975; Weisburger et al., 1969). The similarity in gene expression changes across time, sexes, and studies does imply specificity in a mitogenic MoA for acetamide.

## 5 Conclusions

This study represents a use case scenario for the application of toxicogenomics in the risk assessment of the ubiquitous food contaminant and rodent carcinogen, acetamide. Our approach for the evaluation of acetamide was based on the numerous reports demonstrating that toxicogenomic data can be used to derive PODs which are consistent with apical responses seen in chronic bioassays. Such approaches have significant potential for reducing the time and costs for toxicity testing and health risk assessments while still ensuring protection of human health. Our study strongly supports a mitogenic cancer MoA for acetamide in the rat liver that differs from receptor-mediated mitogenic MoAs of other rodent carcinogens. This conclusion was supported by RNA-seq analysis which identified the enrichment of cell proliferation pathways and the more traditional evaluation of cell proliferation *via* the Ki67 labeling index. While this study has not unequivocally elucidated acetamide's MoA at the molecular level, the consistency of PODs derived for both transcriptional and apical markers suggests that transcriptomic derived PODs can be used to support risk assessment. This was emphasized by the concordance between the functional classification of DEGs, largely associated with cell cycle regulation, and the most sensitive apical endpoint, cell proliferation. Based on the weight of evidence from this study, we estimate a POD of 190 mkd for acetamide-induced rat liver carcinogenesis which may serve as reference point for formal risk assessment.

The following are the supplementary data related to this article.

Supplementary Fig. S1 Frequency distribution of absolute fold changes of genes differentially expressed (|fold change| ≥ 1.5 & FDR ≤ 0.05) in Wistar rats gavaged daily with acetamide. Fold changes from the highest dose group (1500 mkd) was used to calculate frequency, expressed as percent of DEGs. Supplementary Fig. S1

Supplementary Table S1 Raw data for individual rats in experiment A. Supplementary Table S1

Supplementary Table S2 Raw data for individual rats in experiment B. Supplementary Table S2

Supplementary Table S3 List of approaches for the estimation of the toxicogenomic POD. Supplementary Table S3

Supplementary Table S4 RNA-seq fold changes for experiment A. Supplementary Table S4

Supplementary Table S5 RNA-seq fold changes for male rats treated with acetamide for 7 days in experiment B. Supplementary Table S5

Supplementary Table S6 RNA-seq fold changes for male rats treated with acetamide for 28 days in experiment B. Supplementary Table S6

Supplementary Table S7 RNA-seq fold changes for female rats treated with acetamide for 7 days in experiment B. Supplementary Table S7

Supplementary Table S8 RNA-seq fold changes for female rats treated with acetamide for 28 days in experiment B. Supplementary Table S8

## Author contributions

R.N., B.B., F.T., B.G., J.K., T.Z., and V·B., conceived of and designed the experiments. S.K., K.,K., J.K., L.A., and B.P. performed all *in vivo* experiments. S.K., N.P. and N.K. performed sequencing experiments. R.N., M.B., M.A., P.M., and K.T., performed subsequent analysis of RNA-seq data. R.N., B.B., F.T., M.B., M.A., P.M., K.B., B.G., J.K., T.Z., and V.B contributed to the interpretation of the data. The initial manuscript draft was prepared by R.N. and all co-authors reviewed and provided input.

## Declaration of Competing Interest

The authors declare that they have no known competing financial interests or personal relationships that could have appeared to influence the work reported in this paper.

## References

[bb0005] IARC (1999). Re-evaluation of some organic chemicals, hydrazine and hydrogen peroxide. Proceedings of the IARC working group on the evaluation of carcinogenic risks to humans. Lyon, France, 17–24 February 1998. IARC Monographs on the Evaluation of Carcinogenic Risks to Humans.

[bb0010] NTP (2004). A National Toxicology Program for the 21st Century: A Roadmap to Achieve the NTP Vision..

[bb0015] NTP (2018). Research Report on National Toxicology Program Approach to Genomic Dose-response Modeling.

[bb0020] Abbott P.J., Mattia A., Renwick A.J., DiNovi M. (2006). Aliphatic and aromatic amines and amides. Safety Evaluation of Certain Food Additives.

[bb0025] Anders S., Pyl P.T., Huber W. (2015). HTSeq--a Python framework to work with high-throughput sequencing data. Bioinformatics.

[bb0030] Andersen M.E., Clewell H.J., Bermudez E., Willson G.A., Thomas R.S. (2008). Genomic signatures and dose-dependent transitions in nasal epithelial responses to inhaled formaldehyde in the rat. Toxicol. Sci..

[bb0035] Andersen M.E., Clewell H.J., Bermudez E., Dodd D.E., Willson G.A., Campbell J.L., Thomas R.S. (2010). Formaldehyde: integrating dosimetry, cytotoxicity, and genomics to understand dose-dependent transitions for an endogenous compound. Toxicol. Sci..

[bb0040] Bals B., Murnen H., Allen M., Dale B. (2010). Ammonia fiber expansion (AFEX) treatment of eleven different forages: improvements to fiber digestibility in vitro. Anim. Feed Sci. Technol..

[bb0045] Bals B., Teymouri F., Haddad D., Julian W.A., Vismeh R., Jones A.D., Mor P., Van Soest B., Tyagi A., VandeHaar M., Bringi V. (2019). Presence of Acetamide in Milk and beef from cattle consuming AFEX-treated crop residues. J. Agric. Food Chem..

[bb0050] Barton-Maclaren T.S., Westphal M., Sarwar E., Mattison D., Chiu W.A., Dix D., Kavlock R., Krewski D. (2017). Challenges and opportunities in the risk assessment of existing substances in Canada: lessons learned from the international community. Int. J. Risk Assess. Manag..

[bb0055] Bercu J.P., Jolly R.A., Flagella K.M., Baker T.K., Romero P., Stevens J.L. (2010). Toxicogenomics and cancer risk assessment: a framework for key event analysis and dose-response assessment for nongenotoxic carcinogens. Regul. Toxicol. Pharmacol. : RTP.

[bb0060] Bercu J.P., Galloway S.M., Parris P., Teasdale A., Masuda-Herrera M., Dobo K., Heard P., Kenyon M., Nicolette J., Vock E., Ku W., Harvey J., White A., Glowienke S., Martin E.A., Custer L., Jolly R.A., Thybaud V. (2018). Potential impurities in drug substances: compound-specific toxicology limits for 20 synthetic reagents and by-products, and a class-specific toxicology limit for alkyl bromides. Regul. Toxicol. Pharmacol. : RTP.

[bb0065] Bhat V.S., Hester S.D., Nesnow S., Eastmond D.A. (2013). Concordance of transcriptional and apical benchmark dose levels for conazole-induced liver effects in mice. Toxicol. Sci..

[bb0070] Bogen K., Nault R., Bringi V. (2019). Unpublished. PBPK Analysis of acetamide..

[bb0075] Bolger A.M., Lohse M., Usadel B. (2014). Trimmomatic: a flexible trimmer for Illumina sequence data. Bioinformatics.

[bb0080] Chepelev N.L., Moffat I.D., Labib S., Bourdon-Lacombe J., Kuo B., Buick J.K., Lemieux F., Malik A.I., Halappanavar S., Williams A., Yauk C.L. (2015). Integrating toxicogenomics into human health risk assessment: lessons learned from the benzo[a]pyrene case study. Crit. Rev. Toxicol..

[bb0085] Chieli E., Aliboni F., Saviozzi M., Malvaldi G. (1987). Induction of micronucleated erythrocytes by primary thioamides and their metabolites in the mouse. Mutat. Res..

[bb0090] Clewell H.J., Efremenko A., Campbell J.L., Dodd D.E., Thomas R.S. (2014). Transcriptional responses in the rat nasal epithelium following subchronic inhalation of naphthalene vapor. Toxicol. Appl. Pharmacol..

[bb0095] Cohen S.M. (2010). Evaluation of possible carcinogenic risk to humans based on liver tumors in rodent assays: the two-year bioassay is no longer necessary. Toxicol. Pathol..

[bb0100] Cohen S.M., Ellwein L.B. (1990). Cell proliferation in carcinogenesis. Science.

[bb0105] Corton J.C., Cunningham M.L., Hummer B.T., Lau C., Meek B., Peters J.M., Popp J.A., Rhomberg L., Seed J., Klaunig J.E. (2014). Mode of action framework analysis for receptor-mediated toxicity: the peroxisome proliferator-activated receptor alpha (PPARalpha) as a case study. Crit. Rev. Toxicol..

[bb0110] De Boeck M., van der Leede B.J., Van Goethem F., De Smedt A., Steemans M., Lampo A., Vanparys P. (2005). Flow cytometric analysis of micronucleated reticulocytes: time- and dose-dependent response of known mutagens in mice, using multiple blood sampling. Environ. Mol. Mutagen..

[bb0115] Dessau F.I., Jackson B. (1955). Acetamide-induced liver cell alterations in rats. Lab. Invest..

[bb0120] Dragan Y.P., Bidlack W.R., Cohen S.M., Goldsworthy T.L., Hard G.C., Howard P.C., Riley R.T., Voss K.A. (2001). Implications of apoptosis for toxicity, carcinogenicity, and risk assessment: fumonisin B(1) as an example. Toxicol. Sci..

[bb0125] Dybing E., Soderlund E.J., Gordon W.P., Holme J.A., Christensen T., Becher G., Rivedal E., Thorgeirsson S.S. (1987). Studies on the mechanism of acetamide hepatocarcinogenicity. Pharmacol. Toxicol..

[bb0130] EFSA (2014). Modern methodologies and tools for human hazard assessment of chemicals. EFSA J..

[bb0135] Eldridge S.R., Goldsworthy T.L., Popp J.A., Butterworth B.E. (1992). Mitogenic stimulation of hepatocellular proliferation in rodents following 1,4-dichlorobenzene administration. Carcinogenesis.

[bb0140] Elmore S.A., Dixon D., Hailey J.R., Harada T., Herbert R.A., Maronpot R.R., Nolte T., Rehg J.E., Rittinghausen S., Rosol T.J., Satoh H., Vidal J.D., Willard-Mack C.L., Creasy D.M. (2016). Recommendations from the INHAND apoptosis/necrosis working group. Toxicol. Pathol..

[bb0150] Farmahin R., Gannon A.M., Gagne R., Rowan-Carroll A., Kuo B., Williams A., Curran I., Yauk C.L. (2019). Hepatic transcriptional dose-response analysis of male and female Fischer rats exposed to hexabromocyclododecane. Food Chem. Toxicol..

[bb0145] Farmahin R., Williams A., Kuo B., Chepelev N.L., Thomas R.S., Barton-Maclaren T.S., Curran I.H., Nong A., Wade M.G., Yauk C.L. (2017). Recommended approaches in the application of toxicogenomics to derive points of departure for chemical risk assessment. Arch. Toxicol..

[bb0155] Flaks B., Trevan M.T., Flaks A. (1983). An electron microscope study of hepatocellular changes in the rat during chronic treatment with acetamide. Parenchyma, foci and neoplasms. Carcinogenesis.

[bb0160] Fleischman R.W., Baker J.R., Hagopian M., Wade G.G., Hayden D.W., Smith E.R., Weisburger J.H., Weisburger E.K. (1980). Carcinogenesis bioassay of acetamide, hexanamide, adipamide, urea and P-tolylurea in mice and rats. J. Environ. Pathol. Toxicol..

[bb0165] Gitter S. (1956). The influence of acetamide on citrate accumulation after fluoroacetate poisoning. Biochem. J..

[bb0170] Goncharov N.V., Jenkins R.O., Radilov A.S. (2006). Toxicology of fluoroacetate: a review, with possible directions for therapy research. J. Appl. toxicol. : JAT.

[bb0175] Haber L.T., Dourson M.L., Allen B.C., Hertzberg R.C., Parker A., Vincent M.J., Maier A., Boobis A.R. (2018). Benchmark dose (BMD) modeling: current practice, issues, and challenges. Crit. Rev. Toxicol..

[bb0180] Hall A.P., Elcombe C.R., Foster J.R., Harada T., Kaufmann W., Knippel A., Kuttler K., Malarkey D.E., Maronpot R.R., Nishikawa A., Nolte T., Schulte A., Strauss V., York M.J. (2012). Liver hypertrophy: a review of adaptive (adverse and non-adverse) changes--conclusions from the 3rd international ESTP expert workshop. Toxicol. Pathol..

[bb0185] Hardy A., Benford D., Halldorsson T., Jeger M.J., Knutsen K.H., More S., Mortensen A., Naegeli H., Noteborn H., Ockleford C., Ricci A., Rychen G., Silano V., Solecki R., Turck D., Aerts M., Bodin L., Davis A., Edler L., Gundert-Remy U., Sand S., Slob W., Bottex B., Abrahantes J.C., Marques D.C., Kass G., Schlatter J.R. (2017). Update: use of the benchmark dose approach in risk assessment. EFSA J..

[bb0190] Hernandez L.G., van Steeg H., Luijten M., van Benthem J. (2009). Mechanisms of non-genotoxic carcinogens and importance of a weight of evidence approach. Mutat. Res..

[bb0195] Holsapple M.P., Pitot H.C., Cohen S.M., Boobis A.R., Klaunig J.E., Pastoor T., Dellarco V.L., Dragan Y.P. (2006). Mode of action in relevance of rodent liver tumors to human cancer risk. Toxicol. Sci..

[bb0200] Kawamoto T., Ito Y., Morita O., Honda H. (2017). Mechanism-based risk assessment strategy for drug-induced cholestasis using the transcriptional benchmark dose derived by toxicogenomics. J. Toxicol. Sci..

[bb0205] Klaunig J.E., Gehen S.C., Wang Z., Klein P.J., Billington R. (2015). Mechanism of 1,3-dichloropropene-induced rat liver carcinogenesis. Toxicol. Sci..

[bb0210] Kuleshov M.V., Jones M.R., Rouillard A.D., Fernandez N.F., Duan Q., Wang Z., Koplev S., Jenkins S.L., Jagodnik K.M., Lachmann A., McDermott M.G., Monteiro C.D., Gundersen G.W., Ma’ayan A. (2016). Enrichr: a comprehensive gene set enrichment analysis web server 2016 update. Nucleic Acids Res..

[bb0215] Labib S., Williams A., Yauk C.L., Nikota J.K., Wallin H., Vogel U., Halappanavar S. (2016). Nano-risk science: application of toxicogenomics in an adverse outcome pathway framework for risk assessment of multi-walled carbon nanotubes. Part. Fibre Toxicol..

[bb0220] Lachmann A., Xu H., Krishnan J., Berger S.I., Mazloom A.R., Ma'ayan A. (2010). ChEA: transcription factor regulation inferred from integrating genome-wide ChIP-X experiments. Bioinformatics.

[bb0225] Langmead B., Salzberg S.L. (2012). Fast gapped-read alignment with bowtie 2. Nat. Methods.

[bb0230] Love M.I., Huber W., Anders S. (2014). Moderated estimation of fold change and dispersion for RNA-seq data with DESeq2. Genome Biol..

[bb0235] McMullen P.D., Bhattacharya S., Woods C.G., Sun B., Yarborough K., Ross S.M., Miller M.E., McBride M.T., LeCluyse E.L., Clewell R.A., Andersen M.E. (2014). A map of the PPARalpha transcription regulatory network for primary human hepatocytes. Chem. Biol. Interact..

[bb0240] McMullen P.D., Pendse S.N., Black M.B., Mansouri K., Haider S., Andersen M.E., Clewell R.A. (2019). Addressing systematic inconsistencies between in vitro and in vivo transcriptomic mode of action signatures. Toxicol. in Vitro.

[bb0245] Mirkova E.T. (1996). Activities of the rodent carcinogens thioacetamide and acetamide in the mouse bone marrow micronucleus assay. Mutat. Res..

[bb0250] Miura D., Kasahara Y., Morita K., Nakai Y., Hirabayashi K., Wada H., Takahashi Y., Izawa Y., Makita T. (1994). Acetamide induced neither bacterial gene mutations nor micronuclei in mice. MMS Communications.

[bb0255] Moffat I., Chepelev N., Labib S., Bourdon-Lacombe J., Kuo B., Buick J.K., Lemieux F., Williams A., Halappanavar S., Malik A., Luijten M., Aubrecht J., Hyduke D.R., Fornace A.J., Swartz C.D., Recio L., Yauk C.L. (2015). Comparison of toxicogenomics and traditional approaches to inform mode of action and points of departure in human health risk assessment of benzo[a]pyrene in drinking water. Crit. Rev. Toxicol..

[bb0260] Moore M.M., Gollapudi B., Nagane R., Khan N., Patel M., Khanvilkar T., Roy A.M., Ramesh E., Bals B., Teymouri F., Nault R., Bringi V. (2019). The food contaminant acetamide is not an in vivo clastogen, aneugen, or mutagen in rodent hematopoietic tissue. Regul. Toxicol. pharmacol. : RTP.

[bb0265] Morita T., Asano N., Awogi T., Sasaki Y.F., Sato S., Shimada H., Sutou S., Suzuki T., Wakata A., Sofuni T., Hayashi M. (1997). Evaluation of the rodent micronucleus assay in the screening of IARC carcinogens (groups 1, 2A and 2B) the summary report of the 6th collaborative study by CSGMT/JEMS MMS. Collaborative study of the micronucleus group test. Mammalian mutagenicity study group. Mutat. Res..

[bb0270] Pendse S., McMullen P. (2014). An interactive visualization tool to interpret transcriptomic data. Proceedings of the 6th Internation Conference on Bioinformatics and Computational Biology.

[bb0275] Phillips J.R., Svoboda D.L., Tandon A., Patel S., Sedykh A., Mav D., Kuo B., Yauk C.L., Yang L., Thomas R.S., Gift J.S., Davis J.A., Olysyzk L., Merrick B.A., Paules R.S., Parham F., Saddler T., Shah R.R., Auerbach S.S. (2019). BMDExpress 2: enhanced transcriptomic dose-response analysis workflow. Bioinformatics..

[bb0280] Pietrocola F., Galluzzi L., Bravo-San Pedro J.M., Madeo F., Kroemer G. (2015). Acetyl coenzyme a: a central metabolite and second messenger. Cell Metab..

[bb0285] Proudfoot A.T., Bradberry S.M., Vale J.A. (2006). Sodium fluoroacetate poisoning. Toxicol. Rev..

[bb0290] Putcha L., Griffith D.P., Feldman S. (1984). Disposition of 14C-acetohydroxamic acid and 14C-acetamide in the rat. Drug Metab. Dispos..

[bb0295] Slob W. (2017). A general theory of effect size, and its consequences for defining the benchmark response (BMR) for continuous endpoints. Crit. Rev. Toxicol..

[bb2000] Takeda Y., Tominaga T., Tei N., Kitamura M., Taga S. (1975). Inhibitory effect of L-arginine on growth of rat mammary tumors induced by 7,12-dimethylbenz(a)anthracene. Cancer Res.

[bb0300] Thomas R.S., Allen B.C., Nong A., Yang L., Bermudez E., Clewell H.J., Andersen M.E. (2007). A method to integrate benchmark dose estimates with genomic data to assess the functional effects of chemical exposure. Toxicol. Sci..

[bb0305] Thomas R.S., Clewell H.J., Allen B.C., Wesselkamper S.C., Wang N.C., Lambert J.C., Hess-Wilson J.K., Zhao Q.J., Andersen M.E. (2011). Application of transcriptional benchmark dose values in quantitative cancer and noncancer risk assessment. Toxicol. Sci..

[bb0310] Thomas R.S., Clewell H.J., Allen B.C., Yang L., Healy E., Andersen M.E. (2012). Integrating pathway-based transcriptomic data into quantitative chemical risk assessment: a five chemical case study. Mutat. Res..

[bb0315] Thomas R.S., Himmelstein M.W., Clewell H.J., Yang Y., Healy E., Black M.B., Andersen M.E. (2013). Cross-species transcriptomic analysis of mouse and rat lung exposed to chloroprene. Toxicol. Sci..

[bb0320] Thomas R.S., Wesselkamper S.C., Wang N.C., Zhao Q.J., Petersen D.D., Lambert J.C., Cote I., Yang L., Healy E., Black M.B., Clewell H.J., Allen B.C., Andersen M.E. (2013). Temporal concordance between apical and transcriptional points of departure for chemical risk assessment. Toxicol. Sci..

[bb0325] Vismeh R., Haddad D., Moore J., Nielson C., Bals B., Campbell T., Julian A., Teymouri F., Jones A.D., Bringi V. (2018). Exposure assessment of Acetamide in Milk, beef, and coffee using Xanthydrol derivatization and gas chromatography/mass spectrometry. J. Agric. Food Chem..

[bb0330] Wang Z., Neal B.H., Lamb J.C., Klaunig J.E. (2015). Mechanistic investigation of Toxaphene induced mouse liver tumors. Toxicol. Sci..

[bb0335] Wang Z., Li X., Klaunig J.E. (2017). Investigation of the mechanism of triclosan induced mouse liver tumors. Regul. Toxicol. Pharmacol. : RTP.

[bb0340] Wang Z., Li X., Wu Q., Lamb J.C.t., Klaunig J.E. (2017). Toxaphene-induced mouse liver tumorigenesis is mediated by the constitutive androstane receptor. J. Appl. Toxicol. : JAT.

[bb0345] Watanabe T., Suzuki T., Natsume M., Nakajima M., Narumi K., Hamada S., Sakuma T., Koeda A., Oshida K., Miyamoto Y., Maeda A., Hirayama M., Sanada H., Honda H., Ohyama W., Okada E., Fujiishi Y., Sutou S., Tadakuma A., Ishikawa Y., Kido M., Minamiguchi R., Hanahara I., Furihata C. (2012). Discrimination of genotoxic and non-genotoxic hepatocarcinogens by statistical analysis based on gene expression profiling in the mouse liver as determined by quantitative real-time PCR. Mutat. Res..

[bb0350] Webster A.F., Chepelev N., Gagne R., Kuo B., Recio L., Williams A., Yauk C.L. (2015). Impact of genomics platform and statistical filtering on transcriptional benchmark doses (BMD) and multiple approaches for selection of chemical point of departure (PoD). PLoS One.

[bb0355] Weisburger J.H., Yamamoto R.S., Glass R.M., Frankel H.H. (1969). Prevention by arginine glutamate of the carcinogenicity of acetamide in rats. Toxicol. Appl. Pharmacol..

[bb0360] Yauk C.L., Cheung C., Barton-Maclaren T.S., Boucher S., Bourdon-Lacombe J., Chauhan V., Gagné M., Gillespie Z., Halappanavar S., Honeyman M., Jones S.R., Jones-McLean E., Labib S., MacAulay J., Moore J., Paquette M., Petronella N., Semalulu S., Slot A., Vespa A., Woodland C. (2019). Evaluation of the Use of Toxicogenomics in Risk Assessment at Health Canada.

[bb0365] Zhou Y.H., Cichocki J.A., Soldatow V.Y., Scholl E.H., Gallins P.J., Jima D., Yoo H.S., Chiu W.A., Wright F.A., Rusyn I. (2017). Editor's highlight: comparative dose-response analysis of liver and kidney transcriptomic effects of trichloroethylene and tetrachloroethylene in B6C3F1 mouse. Toxicol. Sci..

