# Supplementary material for: A toxicogenomic approach for the risk assessment of the food contaminant acetamide
Source: Toxicol Appl Pharmacol. 2020 Feb 1;388:114872. doi: 10.1016/j.taap.2019.114872 (PMC7014822; doi:10.1016/j.taap.2019.114872)
Supplement: Supplementary Fig. S1 — Frequency distribution of absolute fold changes of genes differentially expressed (|fold change| ≥ 1.5 & FDR ≤ 0.05) in Wistar rats gavaged daily with acetamide. Fold changes from the highest dose group (1500 mkd) was used to calculate frequency, expressed as percent of DEGs. [file mmc1.pdf]

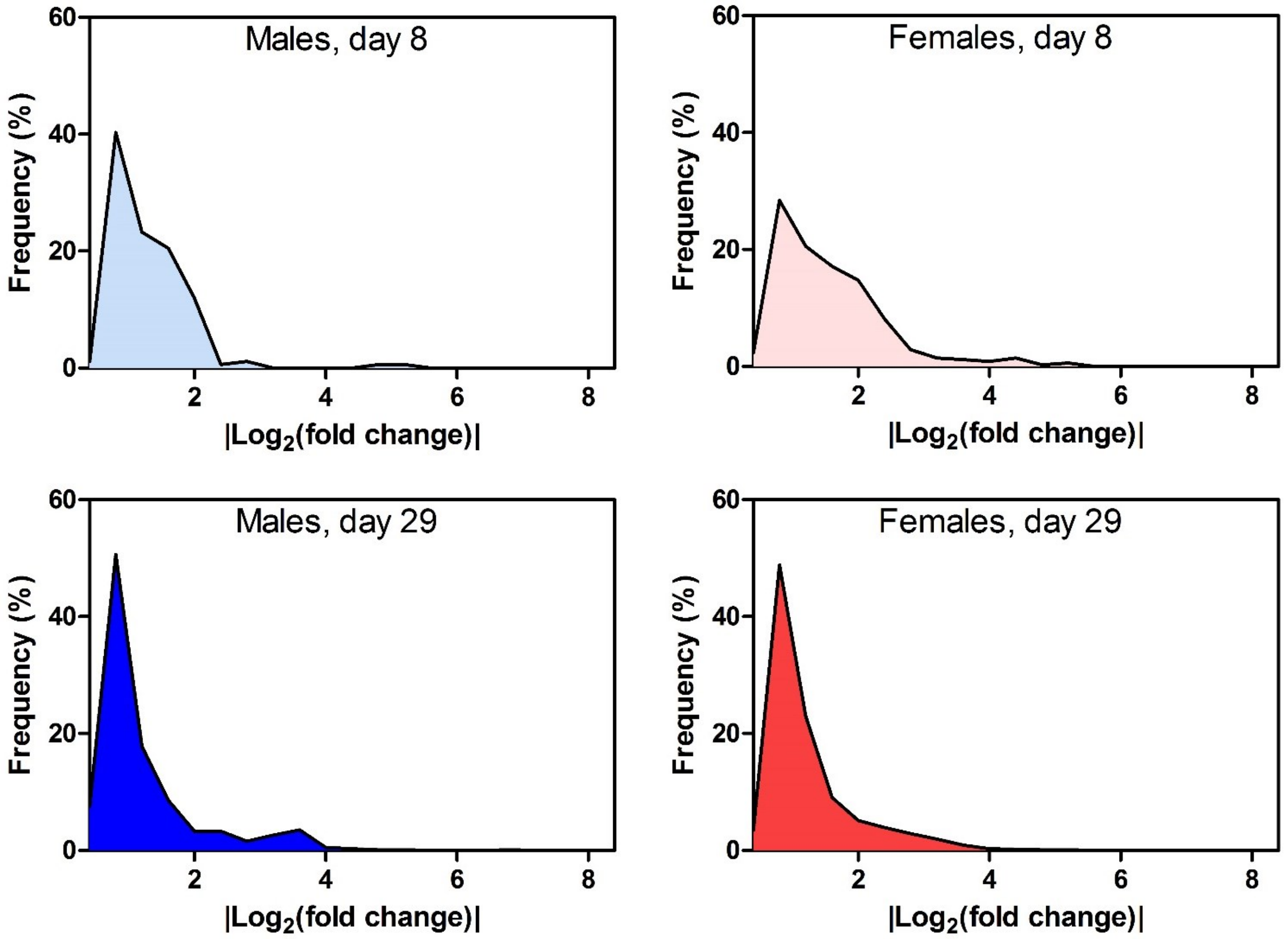

Figure S1. Frequency distribution of absolute fold changes of genes differentially expressed (|fold change| ≥ 1.5 & FDR ≤ 0.05) in Wistar rats gavaged daily with acetamide. Fold changes from the highest dose group (1500 mkd) was used to calculate frequency, expressed as percent of DEGs.
